# Consumers’ Preferences and Willingness to Pay for Fish Products with Health and Environmental Labels: Evidence from Five European Countries

**DOI:** 10.3390/nu12092650

**Published:** 2020-08-31

**Authors:** Davide Menozzi, Thong Tien Nguyen, Giovanni Sogari, Dimitar Taskov, Sterenn Lucas, José Luis Santiago Castro-Rial, Cristina Mora

**Affiliations:** 1 Department of Food and Drug, University of Parma, 43124 Parma, Italy; davide.menozzi@unipr.it (D.M.); giovanni.sogari@unipr.it (G.S.); cristina.mora@unipr.it (C.M.); 2 Truong Dai Hoc Nha Trang, Nr. 02, Nguyen Dinh Chieu, Nha Trang, Khánh Hòa 650000, Vietnam; 3 Institute of Aquaculture University of Stirling, Stirling FK9 4LA, UK; dimitar.taskov@stir.ac.uk; 4 SMART-LERECO AO-INRAE, 35042 Rennes CEDEX, France; sterenn.lucas@agrocampus-ouest.fr; 5 Centro Tecnológico del Mar-Fundación CETMAR, Fisheries Socioeconomic Department, 36208 Vigo, Spain; jsantiago@cetmar.org

**Keywords:** choice experiment, willingness to pay (WTP), consumers’ preferences, sustainability label, nutrition and health claim, fish species

## Abstract

Seafood products are important sources of protein and components of a healthy and sustainable diet. Understanding consumers’ preferences for fish products is crucial for increasing fish consumption. This article reports the consumer preferences and willingness to pay (WTP) for different fish species and attributes on representative samples in five European countries (*n* = 2509): France, Germany, Italy, Spain, and the UK. Consumer choices were investigated for fresh fish in a retail market under hypothetical situations arranged by a labelled choice experiment conducted for seven fish species: Cod, herring, seabass, seabream, salmon, trout, and pangasius. The results show the highest premiums for wild-caught fish than farm-raised alternatives. Ready-to-cook products are generally preferred to whole fish, whereas fish fillet preference is more species-specific. The results show positive premiums for a sustainability label and nutrition and health claims, with high heterogeneity across countries and species. With consumers’ preferences and WTP being largely country- and fish-dependent, businesses (fish companies, retailers, and others) should consider the specific market context and adapt their labelling strategies accordingly. Public authorities campaigns should inform consumers about the tangible benefits related with health and environmental labels.

## 1. Introduction

Due to the increasing number of diet-related chronic diseases and the impactful environmental damage related to food production and consumption, the concept of healthier and more sustainable dietary habits has been strongly promoted both by public authorities and the private sector [1,2,3]. Food policy makers have especially tried to promote such behavior through information on the food label like nutrition and sustainable claims. However, the effectiveness of food policies and labelling strongly depends on the understanding of the complexity of consumer choices and the associated elements [4]. For instance, even if fisheries and aquaculture products are an important source of protein and other beneficial components for human health, such as nutrients and essential long-chain polyunsaturated fatty acids (omega-3 fatty acids), their consumption varies greatly across countries. In the EU, fish and seafood consumption has risen over the past 10 years up to the current 24.3 kg per capita per year, with wide differences between countries, ranging from 5.6 kg in Hungary to 56.8 kg in Portugal [5]. Moreover, dietary recommendations and guidelines for fish consumption differ between the EU countries, both in qualitative and quantitative terms [6]. This might be due to inappropriate and broadly-oriented communication strategies [7], as information campaigns toward a specific target of consumers showed higher impact on food choices [8]. Therefore, in a fish market driven by demand, a better understanding of consumers’ preferences across the European Union (EU) countries for fish species and product attributes is paramount to sustain this sector.

The role of sustainability and nutrition certification and labelling is transmitting information about an intrinsic quality of a product, e.g., relating to public benefits such as environmental integrity, which is not obvious to consumers when choosing a product. The incentive of producers occurs in the form of a premium received from the final consumer and transmitted up the value chain to the producer to cover the increased operating costs of the augmented practices. For evaluating the profitability of newly designed seafood products, a growing number of studies have focused on consumer preferences and attitude toward fish and seafood, as well as on their willingness to pay a premium price for innovative product features.

The nutritional aspects of fish and the related health effects are among the most important factors affecting consumer choices. Concerning the health benefits, the high omega-3 fatty acid and protein contents, as well as the low fat content, are generally associated with the consumer’s perception of fish and seafood as healthy foods [9]. In the past, both qualitative [10] and quantitative [11] studies highlighted the increasing interest in information on the nutritional aspects of fish. However, knowledge about specific nutritional and health benefits of fish consumption does not appear to be strong among the population [9]. Pieniak et al. [7] identified and profiled consumers based on fish consumption, attitudes and knowledge about the health benefits of fish, and the socio-demographic characteristics in three EU countries (France, Poland and Spain). Their results showed that positive health enthusiasts, accounting for only 28% of the sample, have a strong involvement in healthy eating and a higher fish consumption. They also reported the highest subjective knowledge and a higher factual knowledge about fish than consumer segments with a lower interest in healthy eating.

Myrland et al. [12] found that the perception of fish as “difficult to prepare” negatively affected the purchase of whole fish. In this context, new processed fish products (e.g., burgers and ready-to-cook meals) represent an opportunity for producers and retailers to reach those consumers who normally do not buy seafood due to its smell and long preparation time [13]. The importance of the product presentation format is species-related too; for instance, Portuguese consumers seem to prefer whole fish products than fish steaks or fillets, in particular for species like sardine and mackerel that are most often served whole in culinary conventions [14]. Similarly, Thong et al. [15] showed that French consumers are willing to pay a price premium for fillets of pangasius, saithe, and salmon, but not for seabream, sole, tuna, and monkfish. Therefore, the socio-demographic characteristics of consumers and cultural traditions in seafood consumption are likely to influence the ready-meal market development.

The production method, whether wild-caught or farm-raised, is another factor that has been widely investigated. Many studies [16,17,18] indicated that consumers generally prefer wild-caught fish, as they are perceived as being superior in terms of taste, safety, and nutritional value [9,19]. However, other studies reported that preferences for wild-caught or farm-raised alternatives vary across species [15] or combined with other relevant attributes, such as sustainability labels [20,21,22].

Although increasing aquaculture may assist with preventing depletion of wild fish stocks, both wild-caught and farmed fish have substantial environmental impacts [23]. Sustainability labelling is a market-based instrument promoting sustainable fisheries [24,25], considered an incentive for a responsible management of fisheries [26,27] as it decreases the information gap between producers and consumers [28]. Specifically, eco-labels are becoming an important attribute of fish choice, and preferences over eco-labelled seafood products have been studied for wild [20,29,30,31,32,33,34,35,36,37,38,39,40] and farmed species [20,41,42,43,44]. Other authors showed that most consumers associate sustainability labels on food products with aspects of environmental protection rather than ethical issues [4]; this also translates to a lower willingness to pay (WTP) for social benefits of sustainability rather than for ecological benefits [45]. Therefore, as also noticed by Carlucci et al. [9], new insights into consumers’ preferences for sustainable-labelled fish products, and their interaction with other product features, would be useful for producers (fisherpersons, fish mongers, and processors), retailers, and policy makers.

The objective of this study was to investigate consumer demand and choice behavior for fish products in five European countries (France, Germany, Italy, Spain, and the UK). In particular, consumer preferences were examined for different fish species and different attributes, i.e., sustainability label, nutrition and health claims, products presentation, production system and price. A discrete choice experiment (DCE) was applied to accomplish this objective; this method is strongly consistent with the economic demand theory and, in particular, with multi-attribute demand studies based on the Lancastrian consumer theory [46]. The outcomes allowed us to elicit consumers’ preferences and WTP, providing valuable insights into developing targeted food marketing and policies strategies.

## 2. Materials and Methods

### 2.1. Econometric Models

#### 2.1.1. Fish Choice Model

According to Lancaster’s consumer theory [46], consumer utility stems from product attributes, not the products themselves. In other words, consumer utility can be separated into part-worth utilities. The part-worth utilities equal consumers’ preference for corresponding attributes. In marketing research, the product attributes are classified into extrinsic and intrinsic attributes [47,48]. Regardless of whether consumers are exposed to these attributes, they may be important signals of product quality and determinants of consumer preference. The overall utility that a consumer obtains from consuming a fish species *j* (*u_ij_*) can be decomposed into two parts: Observable (*v_ij_*) and unobservable ($\varepsilon_{ij}$). In turn, the observable component of the utility is determined by the consumers’ valuation on products attributes. The utility (*u_ij_*) can be formulated as:

(1) $$u_{ij}=v_{ij}+\varepsilon_{ij}=\;x_{ij}^{\prime }\beta +\varepsilon_{ij}$$

where *i* = 1,…, *N* is the individual consumer *i*; *j* = 1,…, *J*, which is the product *j* among *J* products, $u_{ij}$ is the utility obtained by individual *i* from product *j*; $x_{ij}^{\prime }$ is the product attributes; *β* is the vector of part-worth utility; and $\varepsilon_{ij}$ is the random effect.

It is generally assumed that an individual *i* chooses a product alternative *j* ($y_{ij}$) if the utility derived from this alternative is maximized compared to the other alternatives:

(2) $$y_{ij}=\{\begin{array}{c} 1,if\;u_{ij}\geq max\left(u_{i}\right)\\ 0\;otherwise \end{array}$$

When facing a choice of fish products, consumers assign a random utility to each product alternatives, and select the one with the highest derived utility. Assuming that the stochastic components $\varepsilon_{j}$ have independent and identical distributed (*iid*) forms, the probability of a consumer *i* choosing a fish product *j* ($P\left(y_{ij}=1\right))$ given by the Multinomial Logit (MNL) model [49,50] is expressed:

(3) $$P\left(y_{ij}=1\right)=\frac{exp\left(x_{ij}^{\prime }\beta \right)}{\sum_{j=1}^{J}exp\left(x_{ij}^{\prime }\beta \right)}.$$

The MNL model presented in Equation (3) is the basic choice model and was proven to have several disadvantages, such as assuming the *iid* of the error and assuming the homogeneity of consumers preference. To overcome the limitations of MNL, many advanced discrete choice models have been suggested such as the Mixed Logit (ML) models (random coefficient, scaled-multinomial logit, and generalized-multinomial logit) and the Latent Class model [51,52]. We extended the basic model to mitigate the disadvantages and take advantage of our experimental data. For instance, we applied the Random Price Effect model (i.e., ML model) to evaluate the individual consumer’s preference for the quality attributes of fish. The Mixed Logit (ML) model, in which price is set as a random effect parameter, can be formulated as:

(4) $$P\left(y_{ij}=1\right)=\int_{\beta }\frac{exp\left(x_{ij}^{\prime }\beta \right)}{\sum_{j=1}^{J}exp\left(x_{ij}^{\prime }\beta \right)}f(\beta |\theta )d\beta .$$

In the unconditional probability in Equation (4), the random parameter (e.g., price) β is the individual-specific parameter that has the density function f(β|θ), given the distributional parameter θ. In contrast to the MNL in Equation (3), the ML model is not a closed-form function, so it is unable to be solved. Simulation was used to obtain the parameter coefficients.

#### 2.1.2. Model Specifications

We collected data via a choice experiment, in which each choice set includes several fish products described by commercial fish species name (e.g., cod, salmon, pangasius), production method (i.e., wild-caught vs. farmed fish), presentation (i.e., whole fish/round cut, fillet, or ready-to-cook), nutrition and health claims (i.e., with/without nutrition and health claims), and sustainability label (i.e., with/without sustainability label). The determined component of the utility function $v_{ij}$ in Equation (1) can therefore be elaborated as:

(5) $$v_{ij}=\propto_{j}Species_{j}+\beta_{1}Method_{ij}+\beta_{2}Presentation_{ij}+\beta_{3}Health_{ij}+\beta_{4}Sustain_{ij}+\;\beta_{5i}Price_{ij}.$$

Notice that the random price effect model is estimated so that the price coefficient is an individual specific parameter ($\beta_{5i}$).

We used a labelled choice experiment [15,53] to collect data, which enabled us to estimate a Fish-Species-Specific Effect (FSSE) model to elicit the consumers’ WTP for fish attributes that are specific to particular fish species. The FSSE model (fish *j*) is expressed as:

(6) $$v_{ij}=\propto_{j}Species_{ij}+\beta_{1j}Method_{ij}+\beta_{2j}Presentation_{ij}+\beta_{3j}Health_{ij}+\beta_{4j}Sustain_{ij}+\;\beta_{5j}Price_{ij},$$

where *β* parameters are estimated for part-worth utility of the regarding attribute of the *j*-th fish species. The difference between the models in Equations (5) and (6) is that *β* parameters are estimated specifically for the *j*-th fish species ($\beta_{j}$). The specification of FSSE allowed us to calculate the WTP for considered attributes specific to each of the seven fish species in the choice experiment. We estimated the WTP specific to fish species with expectation that consumers’ preference for fish quality attributes depends on specific species [9].

#### 2.1.3. WTP Estimates

The WTP for a non-monetary attribute is the price premium that consumers are willing to pay for obtaining a desired attribute level. In other words, WTP is a marginal rate of substitution between specific attributes of interest (quality and price attribute). The marginal WTP is calculated by taking the ratio of the derivatives of both the attribute of interest (say an attribute level *A*, e.g., health) and price/cost. In the case of linearity in the attributes, indirect utility specification is given by:

(7) $$WTP_{A}=\frac{\Delta x_{A}}{\Delta x_{p}}=\frac{\frac{\partial v_{ijA}}{\partial x_{A}}}{\frac{\partial v_{ijA}}{\partial x_{p}}}=-\frac{\beta_{A}}{\beta_{p}}.$$

When the random parameter model is applied in which one or two random parameter follows a distribution, the WTP will also follow a distribution and the calculation in Equation (7) is inaccurate. We applied the Delta method [54] to calculate the WTP for each simulation with assumption of normal distribution of the price coefficient. The individual WTP was then calculated as a mean of the sample. Bayesian statistics use the standard error of the mean, also known as the Monte Carlo standard error (MCSE), which takes into account the autocorrelation and correct the standard error by using effective sample size. Assuming that we have *n iid* samples, the mean estimate is $\bar{\mu }$, and $\rho_{k}\left(\mu \right)$ is the lag *k* autocorrelation for *µ*, the MSCE is calculated by:

(8) $$MCSE\left(\bar{\mu }\right)=\frac{1+2\sum_{k=1}^{\infty }\rho_{k}\left(\mu_{i}\right)}{n}.\frac{\sum_{t=1}^{n}{\left(\mu_{i}^{t}-{\bar{\mu }}_{t}\right)}^{2}}{\left(n-1\right)}.$$

The MCSE provides a measurement of the accuracy of the posterior estimates, and small values do not necessarily indicate that you have recovered the true posterior mean [49].

In addition, the MNL with fish-species-specific effect (i.e., the FSSE model) allowed us to calculate the WTP for each of the seven fish species in the choice experiment. We estimated the WTP specific to fish species considering consumers’ preference for fish quality attributes depends in specific species [15]. For instance, consumers may prefer filleted cod to whole fish cod, but they may prefer whole-fish herring to filleted herring. The WTP for an attribute level *A* from the FSSE model in Equation (6) is calculated as:

(9) $$WTP_{Aj}^{}=-\frac{\beta_{Aj}}{\beta_{5j}}$$

where $WTP_{Aj}^{}$ is the price premium paid for obtaining a desired level of attribute *A* (i.e., product with health claim) of fish *j*, and $\beta_{Aj}$ and $\beta_{5j}$ are the estimated coefficients of attribute *A* and price attributes of fish *j*, respectively The WTP for species-specific attribute is calculated straightforward by the ratio of estimated coefficient of species-specific attributes and species-specific price.

We used the SAS procedure BCHOICE to estimate the fish choice models [55]. The procedure is built up by using Bayesian statistics. We estimated different fish choice models and selected those having the convergence of all estimated parameters [55].

### 2.2. Label Choice Experiment

A previous qualitative study was performed with approx. 90 individual in-depth interviews conducted in five countries (France, Germany, Italy, Spain, and the UK) to identify the positive or negative motives, perceptions, associations, and attitudes toward fish/seafood consumption, with a focus on the chosen species: Salmon, trout, seabass, seabream, pangasius, herring and cod [56]. The seven fish species were selected within the EU’s Horizon 2020 Primefish Project, aiming at analyzing the economic sustainability of the European fisheries and aquaculture sectors. The findings of this qualitative work regarding consumers’ perception of the main fish attributes were considered in the development of the choice experiment survey [56]. The final experimental design consisted of five attributes, defined for the seven fish alternatives: Price, production method, presentation, sustainability label, nutrition and health claim (Table 1).

**Figure 1:**
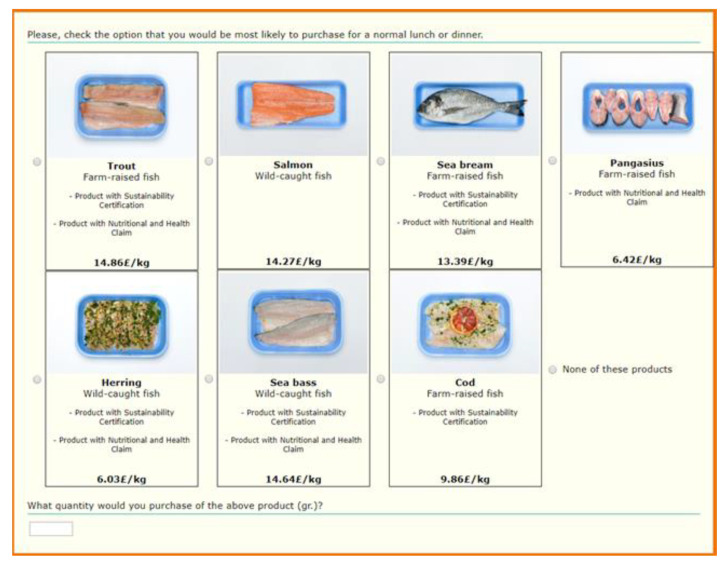
Example of a choice set.

We considered a yearly average market price level (at the retail stage) from official data sources (e.g., governmental agencies, etc.), for the year 2016. The price is indicated in €/kg potentially paid by consumers (£/kg in the U.K.) for the average product/format (fresh product). The production method attribute (wild-caught or farm-raised) was included in the experimental design considering the real availability on the market; in particular, the wild-caught fish level was not applicable for trout and pangasius, whereas the farm-raised fish level was not applicable for herring. The presentation attribute was provided as a picture to consumers. The sustainability label attribute was based on a definition, provided to respondents during the choice experiment as a pop-up linked with the label, based from the available market standards (e.g., Marine Stewardship Council (MSC) and Aquaculture Stewardship Council (ASC); Table 2). Finally, a nutrition and health claim considering the omega-3 fatty acids content and the relative health benefits was used in the experiment (Table 2) [57]. The experimental design resulted in 9 blocks of 8 choice sets with 7 product profiles plus the “no choice” option (Figure 1). Respondents were randomly assigned to 1 of the 9 blocks. We only considered prepacked chilled fresh fish products, and not, e.g., frozen or canned products, to provide respondents with a unique and realistic, although hypothetical, retail context. Chilled fresh fish products are sold separately from, e.g., frozen fish products, which are sold in retail display cabinet used for the sale of frozen foods. The choice experiment was preceded by a cheap talk explaining the rationale behind the experiment and the need to respond carefully to the questions. Cheap talk strategies have been proved to eliminate or reduce hypothetical bias in several estimates [58].

### 2.3. Survey and Data Collection

Data for this study were collected in June 2017 through a nationwide online survey administered in the five countries (France, Germany, Italy, Spain, and the UK) by a third-party contractor using its consumer panel database (the survey questionnaire is available upon request). These five countries represent approx. 73% of the household expenditure on fishery and aquaculture products in Europe [1]. The sample in each country consisted of approximately 500 fish consumers (2509 in total), representative of the national populations in at least three of the following criteria: Age, gender, educational level, and geographical macro-areas (e.g., Italy: North, Center, South). We asked the frequency of fish consumption at the beginning of the questionnaire; answering “never” to this question ended the survey. After removing incomplete questionnaire, a final sample of 2433 consumers was analyzed. The main sample characteristics are reported in Table 3. The regions considered in each country are provided in Table A2.

## 3. Results

Table 4 reports the choice probability of the seven fish species. In general, the results consistently revealed that salmon and cod have the largest market share, exceeding 10% of choice probability in all countries. Salmon and cod are among the most consumed species at the European level and in the surveyed countries [1]. These are followed by seabream and seabass, in particular in Italy, France, and Spain; trout is more likely chosen in Germany and Spain. Even in these countries, the choices expressed in the choice experiment are in line with the actual purchase data [1]. The least frequently chosen fish species were herring and pangasius.

In the Section 3.1 and Section 3.2 we present, respectively, the results of the Mixed Logit (ML) models and the WTP estimates for the considered attributes and levels. In addition, the results of the Fish Species-Specific Effect (FSSE) models and the relative WTP are presented in Table A1 and Table A2, and commented in Section 3.1 and Section 3.2.

### 3.1. Model Estimates

Table 5 reports the mean estimates in all countries for ML models and Monte Carlo standard error (SE); Table A3 provides the results of the FSSE models.

The first relevant observation from the ML model results is the significance of all the coefficients estimated, which led us to conclude that all the selected attributes and levels are relevant to consumers when choosing these fish products. The high *β* coefficient reported in Table 5 for salmon, cod, seabass, and seabream indicated that these fish species are generally preferred by the consumers in the five countries. The results reflected the choices in Table 4, where seabream and seabass were relatively more appreciated in Italy (3.290 and 3.332, respectively) and Spain (3.153 and 3.239, respectively), and trout carried higher utility in Germany (2.877). Salmon showed relatively higher *β* coefficients in all counties. The least preferred species were pangasius and herring. In this latter case, only German and Spanish consumers exhibited a higher coefficient for this species (2.207 and 2.192, respectively). Germany is the only market among those studied where herring is on the top 10 consumed species [1]. In the UK pangasius carried a low but significant effect (0.285), indicating a low utility associated with this fish.

Wild-caught alternatives were generally preferred compared to the farm-raised fish species, as indicated by positive and significant *β* coefficients across all countries, with relatively higher values in France and Italy. When single-species effects were considered (Table A3), a relatively stronger effect was found for seabass (0.406). In single countries, wild seabass and cod were more preferred in Italy (0.449 and 0.482, respectively; Table A3), whereas in France, wild seabream, cod and salmon were more appreciated (0.520, 0.436, and 0.436, respectively). The effect was significant for wild salmon and seabass in the UK (0.284 and 0.431, respectively), and for wild salmon (0.237), seabass (0.569) and seabream (0.269) in Germany; in Spain wild seabass positively affected consumer utility (0.267).

The negative *β* estimates on the presentation row in Table 5 show that ready-to-cook alternatives (the baseline) are generally preferred compared to whole or round-cut fish (–0.118, overall). This was true for almost all countries, with the only exception of Spain, and for all fish species (Table A3). When considering the fish species, round cut salmon was an exception, where a positive estimate (0.253) indicated that this format is preferred over the ready-to-cook alternative; this was also found for whole seabream in Spain (0.238) and Italy (0.179). The fresh fillet was preferred over the ready-to-cook alternative, as indicated in Table 5 by the positive *β* estimate in the first column (0.092), although with heterogeneity across countries. A higher preference for fillets compared to ready-to-cook alternatives was found in France (0.167) and the UK (0.145), whereas lower effects were found in Italy and Germany. When considering species-specific effects, salmon fillet was generally preferred over the ready-to-cook alternative in all countries (0.414; Table A3). This was also noted for cod fillets in France (0.243) and for seabream fillets Spain (0.222) and Italy (0.192). A negative coefficient indicated that ready-to-cook trout was preferred than the fillet format in all countries (–0.191).

The sustainability label was appreciated in all countries considering all species together (0.154; Table 5). This attribute was associated with a higher utility in Germany (0.221) and Italy (0.198). Considering the species-specific effects model, more significant coefficients were found for seabass carrying sustainability label in France and Germany (0.389 and 0.473, respectively; Table A3) and for cod in Italy (0.336). Lower although significant effects were found for seabream in Italy and Germany (0.222 and 0.447, respectively), and for herring in the UK and Germany (0.249 and 0.223, respectively).

The nutrition and health claim estimates indicated a positive effect on consumers‘ utility (0.142), with relatively higher values in Italy and Spain (0.189 and 0.186, respectively). When considering inter-species variability of the estimates, the nutrition and health claim associated with seabass carried higher utility in France and Spain (0.258 and 0.189, respectively; Table A3), whereas seabream with this claim was more appreciated in Italy (0.363) and Germany (0.255). The presence of a nutrition and health claim carried high utility for pangasius in Spain (0.349); for salmon in the UK, Germany, and Spain (0.178, 0.165, and 0.181, respectively); for herring in Germany (0.242); and for trout in Spain (0.198).

### 3.2. WTP Estimates

The WTP results for fish product attributes are shown in Table 6, where the price premiums, expressed in €/kg, obtained from the ML models are reported. Table A4 shows the price premium (in €/kg) per fish species obtained by FSSE Models applying the Equation (9). The WTP values should be interpreted as the maximum amount of money that consumers are willing to pay for obtaining a desired attribute level or, in other words, as the premium that would induce a consumer to be exactly indifferent to buying and not buying fish products with the specified attribute level.

Overall, the highest premium among the attributes proposed was estimated for production method; the ML model for all countries suggested a mean WTP for wild-caught fish relative to farm-raised of €1.29 per kg. The highest premium among the studied countries was found in Italy with €2.03/kg, followed by France (€1.62/kg) and UK (€1.40/kg), whereas the lowest was reported in the Spain (€0.78/kg). A negative premium was found for whole vs. ready-to-cook alternatives, indicating that consumers, on average, were willing to pay an extra premium of €0.50 per kg for a product with convenience (ready-to-cook) over whole format. In this case, the highest premiums were found in Germany (€1.31/kg), and the UK (€1.15/kg). Only Spanish consumers were willing to pay, on average, a €0.25 per kg premium for whole fish species over ready-to-cook alternatives. Considering the product presentation, overall, consumers were willing to pay a €0.43 per kg premium for fillet over ready-to-cook products. The British and French consumers were willing to pay the highest premiums for fish fillets (the estimates were €0.93 and €0.58 per kg, respectively), whereas only German consumers were indifferent (€0.09/kg premium for ready-to-cook over fish fillets).

Considering all countries and all fish species, the consumers were willing to pay a €0.69 per kg premium for fish species with a sustainability label, as estimated by the ML model. This premium was relatively higher in Italy (an average €1.02/kg premium); French and Spanish consumers had lower estimates (€0.43/kg and €0.59/kg, respectively). Finally, consumers were willing to pay a €0.51 per kg premium for fish with nutrition and health claims. Italian and Spanish consumers reported higher premiums in this case, being willing to pay on average €0.96 and €0.92 premiums per kg, respectively.

The WTP estimates from the FSSE models in the different countries revealed the high heterogeneity of the consumers estimates and across different species (Table A4). Focusing on the sustainability label, the highest premiums taking the countries all together were found for herrings and salmon (€2.93 and €1.95 per kg, respectively), whereas lower WTP values were estimated for trout and pangasius (€1.05 and €0.75 per kg, respectively). Considering individual countries, the premiums for the sustainability labels were more relevant for herrings in the UK and Germany; for seabass in Germany and France; and for seabream in Germany and Italy. Other significant effects of the sustainability label attribute were found in Italy for cod, and in France for salmon.

Overall, the highest premiums for the nutrition and health claim were estimated for salmon (€2.65/kg), seabream (€1.21/kg), seabass (€1.15/kg), and cod (€1.12/kg). Lower premiums were found for trout (€0.95/kg) and pangasius (€0.90/kg). The consumer WTP for nutrition and health claim was more relevant in Spain for several species, including pangasius, salmon and trout. The WTP for labelled salmon was also relevant in Germany and the UK Other effects of the nutrition and health label attribute were found in Italy for seabream, in France for seabass and in Germany for herring.

## 4. Discussion and Conclusions

In this study, we investigated consumer choices in five European countries for selected fish attributes: Price, production method, presentation, sustainability label, and nutrition and health claims. Using a labelled hypothetical choice experiment, we estimated their WTP for these attributes across different countries and species, considering consumers’ preferences and willingness to pay for single attributes, e.g., production method, may vary across different cultures and fish species.

First, considering the intrinsic value of the fish species, the model results showed that salmon, cod, seabass, and seabream have highly rank-ordered values in the studied markets, whereas herring, pangasius, and trout received lower evaluations. A familiarity with Mediterranean fish species, such as seabass and seabream, may justify the higher estimates in Italy, Spain, and France [41,42], whereas in Germany and the UK cod and salmon were scored relatively higher. Trout carried higher utility in Germany. These results are consistent with actual consumption data: Salmon and cod are among the most consumed species in the surveyed countries; Italy, Greece, Spain, France, and Portugal have the highest seabass and seabream per capita consumption; herring and trout are among the most consumed fish species in Germany [5]. This data triangulation supports our empirical results.

Our results indicated that respondents are willing to pay more for wild-caught than farm-raised fish. This was verified across all countries and fish species. This result aligns with those of several studies carried out in different countries where wild fish was reported as being perceived as superior to farm-raised fish in terms of taste, safety, and nutritional value [15,16,17,59]. In particular, it was found that when information about the production method was provided to consumers the hedonic evaluation of farmed fish does not change significantly, whereas the liking of wild fish significantly increased [14].

Ready-to-cook products are generally preferred than whole (or round-cut) fish in all countries, with the only exception of Spain. Considering different species, we found consumer preferences for round-cut salmon in France, the UK, and Spain, and whole seabream in Italy and Spain. Salmon and seabream showed the highest choice probability in Spain, and this might have inflated the general preference of Spanish consumers for whole (or round-cut) fish products. Increased familiarity with seabream for Italian and Spanish consumers, increased use with small/medium sized whole products (portion size), and increased familiarity with the cooking skills required for gastronomic preparations might justify the higher WTP for the whole fish. The premiums consumers were willing to pay were generally higher for ready-to-cook pangasius and cod compared to, respectively, round-cut and whole alternatives; ready-to-cook seabass was appreciated in Germany and the UK In this case, the preference for ready-to-cook products might be strictly connected with the desire to save time and effort in food preparation. Convenience perception of meal options is considered an important driver of fish consumption, whereas the difficulty of preparation and manipulation of fish, such as the presence of bones, the lack of cooking skills, and knowledge of recipes for specific fish species, is a strong barrier to the frequency of fish consumption [9,41]. Not surprisingly, round-cut salmon is preferred to the ready-to-cook product since this format is used in several gastronomic preparations.

Fish fillets are generally more appreciated than ready-to-cook alternatives in all countries, with the only exception of Germany where more elaborated products were preferred across fish species. Despite the decline in fish and seafood consumption in Germany in recent years [5], the share of easy-to-cook and convenience fish on the German market is increasing, following a global trend as a consequence of changing lifestyles [60,61]. In the other countries the stated preferences seem more species-specific: Salmon, seabream, and cod fillets are generally preferred to ready-to-cook alternatives, whereas ready-to-cook trout is more appreciated than fillet presentation. In this case, consumers were shown to appreciate new convenient fish products when the original fish characteristics are not significantly altered [14,15]. A French study on seafood showed that increasing the level of processing leads consumers to perceive a decrease in the original quality features like taste, healthiness, and nutritional quality [62]. In Greece, consumers with a high degree of knowledge and expertise in selecting and preparing fish preferred whole or unprocessed fish, whereas younger and less experienced consumers were more willing to consume highly processed and ready-to-cook fish [63].

The results showed positive premiums for a sustainability label, and nutrition and health claims, with high heterogeneity across species and countries. Other studies observed a positive perception and WTP for fish eco-labelling, including specific standards for fishing such as MSC [45], and organic aquaculture [41,42]. However, for sustainability to become a purchase criterion, consumers must have enough information and knowledge about the standard and the requirements, such as resource conservation and depletion of natural fish stocks [20], and trust in the certification system, control mechanisms, and independence of the guarantee body [43]. The WTP estimates are in line with revealed preferences. MSC is the most-studied scheme in fish and seafood using revealed preference methods, and the majority of studies cover the UK retail market for white fish. Sogn-Grundvåg et al. [64] discovered a 10% premium for chilled MSC haddock and 13% premium for MSC cod and haddock in retail market in Glasgow, UK A price premium of 10% was estimated for MSC-certified cod in Sweden [65]. The highest premium for MSC white fish found was 14.2% for frozen Alaska pollock in the London metropolitan area [66]. Similarly, other studies demonstrated consumer interest in the health-related attributes of fish [67], evidencing similarities and differences with those more interested in eco-labelled products [68]. Our results indicated that fish producers could gain significant premiums for sustainability labels as long as they associate benefits to the environment and society with these products [69]. Similarly, the premium that consumers are willing to pay for fish with nutrition and health claims (i.e., an average €0.51 per kg) confirmed the interest in information on the nutritional aspects of fish. However, as noted by other scholars, knowledge about the specific nutritional and health benefits of fish consumption does not appear to be strong among the population [9]. Thus, more evidence of the confidence that consumers attach to the real health benefits provided by fish consumption would help with understanding the real effect of these claims on consumer behavior. The results have also shown different premiums consumers are willing to pay for species with nutrition and health claims, ranging from €0.90/kg (pangasius) to €2.65/kg (salmon). On the one hand, these differences should be considered in relation to the species-specific average prices in the studied countries, as shown in Table A1. For instance, in Italy the premium consumers are willing to pay for pangasius with nutrition and health claims is €0.96/kg, whereas for salmon is €3.19/kg, resulting in a percentage premium above the average market price of, respectively, 17.1% and 21.1%. In France the relative premiums are, respectively, 27.2% and 27.5%. On the other hand, the species-specific variability of the estimates may depend on differences in health benefits perception across species and countries, as also evidenced in other studies [11,18].

The hypothetical nature of the experiment is the main limitation of this study since WTP estimates could have been overestimated; however, the introduction of a cheap talk at the beginning of the choice experiment should have minimized the hypothetical bias [58]. The consistency of our results with the actual consumption patterns in the European countries [5] and with the estimates of revealed preference studies for the sustainability labels indicate the good reliability of the results. Other attributes could have been considered, e.g., country of origin, freshness, and brands, which might have explained consumer choices of fish species and products. For instance, several studies already documented the strong role of domestic origin on consumer choices, which is often perceived as an indicator of the healthfulness and safety of the product [15,41]. Therefore, in this study we decided to focus more on the less-investigated features of fish consumption, at least at the European level, such as sustainability and health-related attributes. The results, obtained in five countries from seven fish species, represent a significant advancement in the understanding of European fish consumer preferences and choices.

In this paper, we provided a wide range of evidence of consumers interest in fish products features from cross-national and cross-species perspectives. Since fish preferences and WTP, for instance for sustainability and production methods, proved to be heterogeneous across countries and species, the results presented in this paper have relevant implications for the success of labelling programs, such as environmental and health-related labelling, applied in different marketing contexts. In other words, businesses (e.g., fish companies and retailers) should consider the specific market context (e.g., familiarity with Mediterranean fish species in Southern EU countries) and adapt their labelling strategies according to country- and species-specific needs. The positive premiums that consumers were willing to pay for eco-friendly and healthy-related labels are promising for public authorities, demonstrating the interest of part of the population in following healthier and more sustainable dietary patterns. The relatively low values of WTP for these attributes in some countries and for some fish species could be due to consumers’ perception of the effectiveness of these attributes, and not by the low interest or value per se. Therefore, the results may also suggest the need to implement homogeneous strategies, within EU countries, for educating consumers about the product labelling and the different claims and certifications which can be found on the pack, and about the tangible benefits to consumers’ related with health and sustainability labels. From the methodological side, further efforts should be devoted to including a measure of beliefs in the choice modelling for improving the understanding of consumers behavior and WTP [69], or by applying other models, such as the Latent Class model, to gather information about different market segments carrying different patterns of preferences and willingness to pay for attributes and fish species.

## Figures and Tables

**Table 1:** Attributes and levels for the choice experiment in the five countries and for the seven fish species (trout, herring, salmon, sea bass, sea bream, cod, and pangasius).

Attributes	Levels
Price	Average market price ^1^ −30% +30%
Production method	Wild-caught fish ^2^ Farm-raised fish ^3^
Presentation (picture)	Whole fish/Round cut ^4^ Fillet Ready to cook
Sustainability label	No Yes
Nutrition and Health Claim	No Yes

^1^ The average market prices are provided in Table A1; ^2^ The wild-caught fish level was not applicable for trout and pangasius; ^3^ The farm-raised fish level was not applicable for herring; ^4^ Round-cut was applied for salmon and pangasius, whole fish for the other species.

**Table 2:** Sustainability label and Nutrition and Health Claim used in the experiment.

Sustainability Label	Nutrition and Health Claim
When labelled according to a sustainability scheme, any fish can be traced back to a fishery or to a fish farm that meets principles reflecting the maintenance and re-establishment of healthy populations of targeted species, the maintenance of the integrity of ecosystems, the use of feed and other inputs that are sourced responsibly, and the social responsibility for workers and communities impacted by fishing and fish farming. This standard is intended to be used on a global basis by accredited third party certifiers to undertake the certification of fisheries and fish farmers to the above mentioned principles and criteria.	Product high in omega-3 fatty acids which contribute to maintenance of normal function of the heart and normal blood pressure, with the following condition of use: The beneficial effect is obtained with a daily intake of 250 mg of omega-3 fatty acids. Such amount can be consumed as part of a balanced diet [57].

**Table 3:** Sample (S) and national census (C) socio-demographic characteristics.

	France		Germany		Italy		Spain		UK		Total
	S	C ^1^	S	C	S	C	S	C	S	C	S
**Number**	485	66.6	485	82.8	494	60.7	496	46.6	473	65.7	2433
**Gender (%)**											
Male	50.7	50.8	52.8	50.7	49.6	49.7	52.2	50.2	50.7	49.8	51.2
Female	49.3	49.2	47.2	49.3	50.4	50.3	47.8	49.8	49.3	50.2	48.8
**Age in years (%)**											
18–24	12.6	13.4	10.9	12.1	10.3	11.0	11.3	10.5	10.4	14.5	11.1
25–34	18.1	20.1	20.2	20.1	19.2	17.9	19.6	19.0	24.1	21.9	20.2
35–44	22.5	21.4	18.8	18.9	23.1	23.3	26.0	26.0	21.4	20.6	22.4
45–54	23.1	22.4	26.4	25.7	25.3	25.5	22.6	24.0	23.0	22.7	24.1
55+	23.7	22.8	23.7	23.3	22.1	22.3	20.6	20.5	21.1	20.3	22.2
**Education (%)**											
Less than lower secondary education	17.9	21.2	16.7	16.0	39.3	38.9	36.1	41.0	15.4	19.8	25.2
Upper secondary, non-tertiary education	47.2	46.0	56.9	58.4	44.4	44.7	27.8	25.4	43.4	40.5	43.8
Tertiary education	34.8	32.8	26.4	25.6	16.4	16.4	36.1	33.7	41.3	39.7	31.0

^1^ Country population in million residents (Eurostat statistics 2016).

**Table 4:** Choice probability obtained by Mixed Logit (ML) models (M, mean; SE, Monte Carlo standard error).

	All Countries		France		Germany		Italy		Spain		UK	
	M	SE	M	SE	M	SE	M	SE	M	SE	M	SE
Cod	0.154	0.001	0.150	0.002	0.235	0.004	0.158	0.001	0.130	0.002	0.235	0.004
Herring	0.088	0.001	0.086	0.001	0.112	0.003	0.070	0.001	0.053	0.001	0.112	0.003
Pangasius	0.082	0.001	0.062	0.001	0.032	0.000	0.076	0.002	0.095	0.002	0.032	0.000
Salmon	0.230	0.002	0.252	0.004	0.280	0.004	0.174	0.004	0.189	0.004	0.280	0.004
Seabass	0.113	0.001	0.107	0.001	0.075	0.003	0.174	0.001	0.136	0.001	0.075	0.003
Seabream	0.119	0.001	0.125	0.001	0.042	0.001	0.198	0.001	0.169	0.001	0.042	0.001
Trout	0.109	0.001	0.104	0.001	0.048	0.001	0.082	0.001	0.156	0.003	0.048	0.001
No choice	0.105	0.003	0.114	0.007	0.176	0.009	0.067	0.005	0.073	0.005	0.176	0.009

**Table 5:** *β* coefficients estimates of Mixed Logit (ML) models (Mean; SE, Monte Carlo standard error).

Species and Attributes	All Countries (*n* = 2433)		France (*n* = 485)		Germany (*n* = 485)		Italy (*n* = 494)		Spain (*n* = 496)		UK (*n* = 473)	
	Mean	SE	Mean	SE	Mean	SE	Mean	SE	Mean	SE	Mean	SE
Cod	2.791	0.002	2.897	0.006	2.641	0.006	3.223	0.005	3.197	0.005	2.774	0.007
Herring	1.381	0.002	1.582	0.005	2.207	0.005	1.963	0.005	2.192	0.006	0.524	0.003
Pangasius	1.285	0.001	1.477	0.004	1.791	0.003	1.643	0.004	1.841	0.004	0.285	0.005
Salmon	3.274	0.002	3.458	0.006	3.626	0.006	3.491	0.006	3.653	0.006	2.979	0.007
Seabass	2.505	0.002	2.505	0.006	2.306	0.006	3.290	0.005	3.153	0.005	1.892	0.008
Seabream	2.437	0.002	2.400	0.005	2.140	0.006	3.332	0.005	3.239	0.005	1.259	0.008
Trout	2.228	0.002	2.586	0.005	2.877	0.005	2.598	0.005	2.552	0.004	1.322	0.007
Wild-Caught vs. Farm-Raised	0.301	0.000	0.455	0.001	0.299	0.001	0.386	0.001	0.157	0.001	0.226	0.001
Presentation: Whole ^1^ vs. Ready-to-Cook	−0.118	0.000	−0.131	0.001	−0.346	0.001	−0.033	0.001	0.052	0.001	−0.174	0.001
Presentation: Fillet vs. Ready-to-Cook	0.092	0.000	0.167	0.001	−0.006	0.001	0.074	0.001	0.108	0.001	0.145	0.001
Sustainability Label	0.154	0.000	0.138	0.001	0.221	0.001	0.198	0.001	0.121	0.001	0.112	0.001
Nutrition and Health Claim	0.142	0.000	0.067	0.001	0.162	0.001	0.189	0.001	0.186	0.001	0.097	0.001
Price (mean)	−0.240	0.003	−0.259	0.004	−0.236	0.007	−0.278	0.008	−0.308	0.010	−0.256	0.009
Price (variance)	0.027	0.000	0.039	0.000	0.036	0.000	0.044	0.000	0.049	0.000	0.039	0.000
Mean of Log-Likelihood	−34,300.30		−6808.12		−6704.31		−7011.89		−7121.84		−5863.98	
Accepted Rate	0.96		0.89		0.92		0.91		0.93		0.89	
Hit probability	0.22		0.23		0.24		0.22		0.21		0.30	
Average Efficiency	0.57		0.56		0.55		0.56		0.57		0.49	

Note: All estimates are significant at *p* < 0.001. ^1^ Round cut for salmon and pangasius, whole fish for the other species.

**Table 6:** Willingness to pay (WTP) estimates in €/kg (mean values, Monte Carlo standard error in parenthesis) of Mixed Logit (ML) models.

Species and Attributes	All Countries (*n* = 2433)	France (*n* = 485)	Germany (*n* = 485)	Italy (*n* = 494)	Spain (*n* = 496)	UK ^1^ (*n* = 473)
Wild-Caught vs. Farm-Raised	1.29 (0.006)	1.62 (0.005)	1.10 (0.007)	2.03 (0.003)	0.78 (0.001)	1.40 (0.002)
Presentation (Whole vs. Ready-to-Cook)	−0.50 (0.002)	−0.43 (0.002)	−1.31 (0.008)	−0.13 (0.001)	0.25 (0.001)	−1.15 (0.002)
Presentation (Fillet vs. Ready-to-Cook)	0.43 (0.002)	0.58 (0.002)	−0.09 (0.001)	0.40 (0.001)	0.50 (0.001)	0.93 (0.002)
Sustainability Label	0.69 (0.003)	0.43 (0.002)	0.60 (0.008)	1.02 (0.002)	0.59 (0.001)	0.75 (0.002)
Nutrition and Health Claim	0.51 (0.003)	0.18 (0.001)	0.42 (0.006)	0.96 (0.002)	0.92 (0.001)	0.65 (0.001)

Note: All estimates are significant at *p* < 0.001. ^1^ The exchange rate used in the UK case was £1 GB = €1.16.

## Appendix A

**Table A1:** Average price levels (€/kg, and £/kg for the UK) by fish species in each country.

Country	Trout	Herring	Salmon	Seabream	Seabass	Cod	Pangasius
France	12.80	9.90	14.90	11.50	14.30	14.90	8.50
Germany	11.58	10.86	16.84	16.70	16.80	16.75	5.25
Italy	10.51	9.90	15.10	10.82	11.82	12.21	5.60
Spain	5.97	11.90	12.87	9.87	11.04	12.00	5.23
UK (€/kg)	16.79	5.24	16.13	21.61	23.64	15.91	10.37
UK (£/kg) ^1^	14.86	4.64	14.27	19.12	20.92	14.08	9.18

^1^ The figure in €/kg was translated in £/kg in the UK. The exchange rate used was £1 GB = €1.16.

**Table A2:** Sample (S) and national census (C) by Region (in %).

France			Germany			Italy			Spain			UK		
Regions	S	C	Regions	S	C	Regions	S	C	Regions	S	C	Regions	S	C
Île de France	19.8	19.7	Baden-Württemb.	14.3	13.7	Nord-Ovest	20.0	26.2	Noroeste	10.8	9.7	North East	4.2	4.0
Bassin Parisien	9.6	16.5	Bayern	16.1	16.2	Nord-Est	23.8	19.0	Noreste	11.2	9.8	North West	11.0	10.9
Nord-Pas-de-Calais	7.0	6.4	Berlin	4.8	4.5	Centro	26.0	19.8	Comunidad de Madrid	16.6	14.7	Yorkshire and The Humber	8.2	8.2
Est	10.2	8.5	Brandenburg	2.6	3.1	Sud	18.8	23.7	Centro	13.2	12.4	East Midlands	7.8	7.1
Ouest	15.2	13.3	Bremen	0.6	0.8	Isole	11.3	11.3	Este	24.4	30.4	West Midlands	8.6	8.7
Sud-Ouest	11.8	11.0	Hamburg	2.4	2.3				Sur	24.0	23.0	East of England	8.2	9.1
Centre-Est	14.0	12.3	Hessen	7.0	7.8							London	16.4	14.4
Méditerranée	12.6	12.3	Mecklenburg-Vorpommern	2.2	2.0							South East	14.0	13.5
			Niedersachsen	10.2	9.8							South West	8.2	8.1
			Nordrhein-Westf.	23.3	22.3							Wales	4.4	4.6
			Rheinland-Pfalz	4.2	5.1							Scotland	7.4	8.4
			Saarland	1.0	1.2							Northern Ireland	1.8	2.8
			Sachsen	5.0	4.9									
			Sachsen-Anhalt	3.0	2.7									
			Schleswig-Holstein	3.4	3.5									

**Table A3:** *β* coefficients estimates (M, mean; SD, standard deviation) of the Fish-Species-Specific Effect (FSSE) model per country.

Attributes/Species Effects	All Countries (*n* = 2433)		France (*n* = 485)		Germany (*n* = 485)		Italy (*n* = 494)		Spain (*n* = 496)		UK (*n* = 473)	
	M	SD	M	SD	M	SD	M	SD	M	SD	M	SD
Species												
Cod	2.073	0.105	2.176	0.244	1.919	0.286	2.558	0.249	2.144	0.262	1.469	0.216
Herring	0.255	0.089	0.600	0.273	1.396	0.237	0.660	0.295	1.099	0.337	−0.064	0.244
Pangasius	1.097	0.097	0.814	0.296	0.812	0.215	1.006	0.278	0.615	0.256	−0.938	0.411
Salmon	1.609	0.095	1.823	0.214	1.761	0.215	1.765	0.239	1.597	0.237	1.569	0.209
Seabass	2.246	0.117	2.040	0.297	1.576	0.360	2.831	0.243	2.325	0.268	0.751	0.359
Seabream	2.303	0.110	2.279	0.273	0.473	0.378	2.367	0.227	2.249	0.241	−0.316	0.433
Trout	1.394	0.083	1.406	0.249	1.613	0.209	1.543	0.267	1.192	0.209	0.245	0.346
Price												
Cod	−0.099	0.005	−0.088	0.013	−0.095	0.013	−0.101	0.015	−0.129	0.017	−0.076	0.011
Herring	−0.053	0.008	−0.082	0.025	−0.123	0.020	−0.071	0.026	−0.137	0.026	−0.086	0.045
Pangasius	−0.213	0.013	−0.150	0.033	−0.068	0.036	−0.188	0.045	−0.099	0.043	−0.073	0.042
Salmon	−0.051	0.005	−0.050	0.011	−0.040	0.009	−0.046	0.012	−0.070	0.013	−0.077	0.011
Seabass	−0.116	0.005	−0.139	0.016	−0.067	0.016	−0.111	0.015	−0.129	0.018	−0.043	0.012
Seabream	−0.164	0.006	−0.132	0.019	−0.051	0.017	−0.125	0.016	−0.164	0.019	−0.055	0.018
Trout	−0.129	0.006	−0.123	0.018	−0.098	0.016	−0.135	0.023	−0.090	0.030	−0.099	0.022
Production Method (Wild-Caught vs. Farm-Raised)												
Cod	0.259	0.041	0.436	0.095	0.205	0.111	0.482	0.091	0.132	0.096	0.084	0.082
Salmon	0.282	0.034	0.436	0.075	0.237	0.075	0.340	0.083	0.105	0.081	0.284	0.075
Seabass	0.406	0.047	0.379	0.105	0.569	0.127	0.449	0.084	0.267	0.093	0.431	0.127
Seabream	0.260	0.046	0.520	0.101	0.269	0.134	0.272	0.081	0.113	0.086	0.111	0.160
Cod	−0.405	0.053	−0.468	0.119	−0.796	0.147	−0.372	0.112	−0.143	0.118	−0.402	0.099
Herring	−0.257	0.064	−0.265	0.151	−0.379	0.127	−0.119	0.159	−0.061	0.185	−0.322	0.129
Pangasius	−0.451	0.068	−0.914	0.187	−0.664	0.120	−0.228	0.154	−0.185	0.135	−0.231	0.228
Salmon	0.253	0.043	0.362	0.094	0.031	0.091	0.266	0.109	0.299	0.104	0.331	0.090
Seabass	−0.150	0.057	−0.078	0.134	−0.506	0.163	−0.041	0.103	0.011	0.115	−0.434	0.157
Seabream	0.041	0.057	−0.127	0.124	−0.213	0.169	0.179	0.101	0.238	0.108	−0.313	0.215
Trout	−0.158	0.056	−0.262	0.132	−0.190	0.110	−0.066	0.136	−0.046	0.104	−0.489	0.194
Presentation (Fillet vs. Ready-to-Cook) ^1^												
Cod	0.109	0.047	0.243	0.104	−0.015	0.122	0.118	0.103	0.120	0.114	0.091	0.090
Herring	−0.022	0.061	0.040	0.137	−0.063	0.118	−0.037	0.155	0.136	0.178	−0.087	0.121
Pangasius	−0.106	0.062	−0.205	0.149	−0.071	0.103	−0.037	0.144	−0.164	0.132	−0.142	0.218
Salmon	0.414	0.043	0.434	0.092	0.253	0.090	0.496	0.105	0.472	0.104	0.479	0.089
Seabass	−0.052	0.055	0.063	0.129	−0.102	0.145	−0.171	0.105	0.030	0.114	−0.027	0.141
Seabream	0.149	0.056	0.083	0.120	−0.040	0.162	0.192	0.103	0.222	0.110	0.149	0.193
Trout	−0.191	0.057	−0.011	0.127	−0.178	0.111	−0.393	0.148	−0.253	0.112	−0.165	0.173
Sustainability Label												
Cod	0.152	0.041	0.017	0.094	0.153	0.111	0.336	0.092	0.153	0.097	0.124	0.080
Herring	0.154	0.052	−0.068	0.119	0.223	0.105	0.144	0.126	0.174	0.148	0.249	0.108
Pangasius	0.159	0.052	0.220	0.136	0.173	0.094	0.221	0.121	0.156	0.108	−0.204	0.186
Salmon	0.100	0.036	0.171	0.077	0.105	0.077	0.066	0.088	0.064	0.086	0.073	0.076
Seabass	0.148	0.046	0.389	0.105	0.473	0.130	0.095	0.085	−0.069	0.093	0.032	0.120
Seabream	0.192	0.045	0.059	0.099	0.447	0.138	0.222	0.081	0.162	0.086	0.165	0.162
Trout	0.135	0.047	0.162	0.108	0.106	0.092	0.200	0.119	0.176	0.091	−0.018	0.152
Cod	0.111	0.040	0.095	0.093	0.180	0.109	0.173	0.088	0.096	0.096	0.054	0.079
Herring	0.082	0.051	0.014	0.116	0.242	0.102	0.081	0.125	0.023	0.142	0.003	0.108
Pangasius	0.191	0.053	−0.009	0.132	0.157	0.091	0.180	0.120	0.349	0.109	0.176	0.181
Salmon	0.136	0.034	−0.010	0.074	0.165	0.073	0.148	0.084	0.181	0.080	0.178	0.071
Seabass	0.134	0.046	0.258	0.107	−0.052	0.123	0.153	0.087	0.189	0.095	0.034	0.122
Seabream	0.197	0.046	0.036	0.098	0.255	0.136	0.363	0.082	0.174	0.086	0.027	0.166
Trout	0.122	0.048	0.027	0.110	0.105	0.091	0.105	0.120	0.198	0.091	0.139	0.155
*Mean of Log-likelihood*	*−37,851.47*		*−7509.23*		*−7529.17*		*−7666.7*		*−7820.090*		*−6818.34*	
*Accepted Rate*	*0.82*		*0.602*		*0.575*		*0.626*		*0.621*		*0.437*	
*Hit probability*	*0.162*		*0.167*		*0.164*		*0.165*		*0.154*		*0.201*	
*Average Efficiency*	*0.935*		*0.647*		*0.598*		*0.71*		*0.696*		*0.372*	

^1^ Round cut for salmon and pangasius, whole fish for the other species.

**Table A4:** Willingness to pay (WTP) estimates (mean values, €/kg) of the Fish Species-Specific Effect (FSSE) model per country.

	All Countries (*n* = 2433)	France (*n* = 485)	Germany (*n* = 485)	Italy (*n* = 494)	Spain (*n* = 496)	UK (*n* = 473)
**Production Method (Wild-Caught vs. Farm-Raised)**	€/kg	€/kg	€/kg	€/kg	€/kg	€/kg
Cod	2.60	4.93	2.15	4.78	1.03	1.10
Salmon	5.50	8.69	5.91	7.33	1.50	3.69
Seabass	3.50	2.72	8.51	4.03	2.06	10.09
Seabream	1.59	3.94	5.29	2.19	0.69	2.03
**Presentation (Whole vs. Ready-to-Cook) ^1^**	€/kg	€/kg	€/kg	€/kg	€/kg	€/kg
Cod	−4.08	−5.29	−8.36	−3.68	−1.11	−5.26
Herring	−4.90	−3.21	−3.08	−1.66	−0.45	−3.75
Pangasius	−2.12	−6.11	−9.77	−1.22	−1.87	−3.15
Salmon	4.93	7.21	0.77	5.72	4.30	4.30
Seabass	−1.29	−0.56	−7.56	−0.37	0.09	−10.16
Seabream	0.25	−0.96	−4.20	1.44	1.45	−5.70
Trout	−1.23	−2.13	−1.93	−0.49	−0.51	−4.94
**Presentation (Fillet vs. Ready-to-Cook)**	€/kg	€/kg	€/kg	€/kg	€/kg	€/kg
Cod	1.09	2.75	−0.15	1.17	0.93	1.19
Herring	−0.42	0.48	−0.51	−0.52	0.99	−1.01
Pangasius	−0.50	−1.37	−1.04	−0.20	−1.66	−1.94
Salmon	8.07	8.65	6.31	10.69	6.78	6.22
Seabass	−0.45	0.46	−1.53	−1.53	0.23	−0.63
Seabream	0.91	0.63	−0.79	1.54	1.35	2.72
Trout	−1.49	−0.09	−1.81	−2.92	−2.81	−1.67
**Sustainability Label**	€/kg	€/kg	€/kg	€/kg	€/kg	€/kg
Cod	1.53	0.19	1.61	3.32	1.18	1.62
Herring	2.93	−0.82	1.81	2.02	1.27	2.89
Pangasius	0.75	1.47	2.55	1.18	1.58	−2.79
Salmon	1.95	3.40	2.61	1.42	0.91	0.95
Seabass	1.27	2.80	7.06	0.86	−0.53	0.75
Seabream	1.17	0.44	8.80	1.78	0.99	3.00
Trout	1.05	1.31	1.08	1.49	1.96	−0.19
**Nutrition and Health Claim**	€/kg	€/kg	€/kg	€/kg	€/kg	€/kg
Cod	1.12	1.07	1.89	1.71	0.74	0.70
Herring	1.56	0.16	1.97	1.14	0.17	0.04
Pangasius	0.90	−0.06	2.31	0.96	3.53	2.41
Salmon	2.65	−0.20	4.10	3.19	2.59	2.31
Seabass	1.15	1.86	−0.78	1.38	1.46	0.80
Seabream	1.21	0.27	5.02	2.91	1.06	0.49
Trout	0.95	0.22	1.06	0.78	2.20	1.40

^1^ Round cut for salmon and pangasius, whole fish for the other species.
